# Methodology and measurement properties of health-related quality of life instruments: A prospective study of patients undergoing breast reduction surgery

**DOI:** 10.1186/1477-7525-3-44

**Published:** 2005-07-22

**Authors:** Achilleas Thoma, Sheila Sprague, Karen Veltri, Eric Duku, William Furlong

**Affiliations:** 1 Department of Surgery, Division of Plastic and Reconstructive Surgery, St. Joseph's Healthcare, Hamilton, Ontario, Canada; 2 Surgical Outcomes Research Centre (SOURCE), McMaster University, Hamilton, Ontario, Canada; 3 Department of Psychiatry and Behavioural Neurosciences, McMaster University, Hamilton, Ontario, Canada; 4 Centre for Health Economics and Policy Analysis, Department of Clinical Epidemiology and Biostatistics, McMaster University, Hamilton, Ontario, Canada; 5 Health Utilities Inc, Dundas, Ontario, Canada

**Keywords:** Breast Reduction Surgery, Health-Related Quality of Life, Reliability, Responsiveness, Validity

## Abstract

**Background:**

Breast hypertrophy is associated with clinically important morbidity. A prospective study was conducted to assess the change in health-related quality of life (HRQL) following breast reduction mammoplasty. This paper describes the measurement properties of each of the HRQL questionnaires used.

**Methods:**

The reliability, responsiveness, and the construct validity of each HRQL instrument (the Health Utilities Index Mark 2 (HUI2) and Mark 3 (HUI3) and the Breast Reduction Assessment Value and Outcomes (BRAVO) instruments) were assessed. The BRAVO instruments are a set of separate instruments including the Short Form-36 (SF-36), the Multidimensional Body Self Relations Questionnaire Appearance Assessment (MBSRQ-AS), and the Breast Related Symptoms Questionnaire (BRSQ).

**Results:**

The HUI2, the HUI3, the MBSRQ-AS, and the breast severity symptom (BSS) score from the BRSQ all demonstrated good test-retest reliability. The SF-36 physical component summary, the MBSRQ-AS, and the BSS score demonstrated high responsiveness. The SF-36 mental component summary and the HUI3 had a moderate effect size and the HUI2 had a small effect size. All of the changes in scales are correlated in the same direction except for the SF-36 physical component summary and the SF-36 mental component summary.

**Conclusion:**

All four instruments were found to be reliable and responsive. These instruments can be used in similar clinical settings to evaluate the change in patients' HRQL.

## Background

Within the last decade the plastic surgical community has been encouraged to use health-related quality of life (HRQL) assessment instruments to report on the efficacy of surgical interventions [1-5]. There is also an increased awareness of the impact of health and healthcare on the quality of human life such as a patient's ability to perform daily activities. Positive themes of happiness, social well-being, and emotional well-being need to be measured as these variables are particularly relevant to plastic surgery. Various HRQL instruments, generic and disease or condition specific, have been applied to plastic surgery research, especially in the area of breast hypertrophy and reduction mammoplasty [6-19]. Evidence from other clinical settings has shown that the generic instruments may be as efficient as the disease-specific ones [20-22]. A recommendation was made by Guyatt et al to include both a generic and a disease (condition) specific instrument in the evaluation of medical interventions [23].

Breast hypertrophy has been reported by patients to be associated with important burdens in pain and discomfort as well as emotion [7]. Earlier breast studies used a variety of study designs, instruments, and outcome measures [6-19]. These studies found that breast hypertrophy was associated with significant morbidity and reduced HRQL. They also found that after breast reduction mammoplasty patients had a substantial improvement in HRQL. Kerrigan et al found that patients with breast hypertrophy had lower health utility scores compared to controls without breast hypertrophy [6]. In a second report, Kerrigan et al found that patients with breast hypertrophy scored lower on the EuroQol; McGill Pain Questionnaire, Multidimensional Body Self Relations Questionnaire (MBSRQ), Short Form 36 (SF-36), and breast-related symptoms questionnaire (BRSQ) than the controls [7]. A recent prospective study found that pre-operatively mammoplasty patients scored lower on the SF-36 compared to normative data and there was an improvement in SF-36 scores from pre-operative to post-operative and these improvements were maintained to 12 months [13]. The improvements noted after the reduction mammoplasty remained stable at three years post-surgery [14]. In a cohort study, Collins et al found that pre-surgery patients scored significantly lower on the SF-36 than normative data and that following reduction mammoplasty patients improved from pre-surgery in all eight domains of the SF-36 [8]. Collins et al also found that post-surgery pain was lower and that the benefits from breast reduction were not associated with body weight, bra cup size, or weight of tissue resection [8].

In a recent Canadian prospective study of patients with a body mass index (BMI) below 27, pre-surgery mammoplasty patients scored lower on the SF-36 compared to normative data and post-surgery these patients achieved scores similar to normative data [18]. Although several publications have addressed HRQL in patients with breast hypertrophy, reduction mammoplasty remains a controversial surgery because of the denial of insurance coverage based on BMI in certain jurisdictions [18,19].

A number of different instruments have been used in previous studies to measure HRQL in patients with breast hypertrophy. In terms of the hierarchy of evidence in surgical studies, the studies which provide the higher strength of evidence are prospective cohort studies which address important patient outcomes. These studies have shown an improvement from pre-operative to post-operative, which have been statistically significant. Our study is similar to the design of some of the earlier prospective cohort studies measuring HRQL in patients with breast hypertrophy [8,10,13-15,18]. A recent study and discussion by Kerrigan et al stresses the importance of measuring HRQL and incorporating patient-reported health status into everyday practice [18,24]. The current study is the first to use the Health Utilities Index (HUI) as an outcome assessment [25-28]. This study is also the first prospective study to simultaneously assess the measurement properties of four HRQL instruments in breast reduction patients.

The primary objective of this study is to look at the measurement properties, including the reliability and responsiveness, of each of the four HRQL instruments used. The secondary objective was to assess the concurrent validity of each of the four HRQL instruments.

## Methods

### Patient eligibility and study design

Consecutive patients seen by the senior author (AT) over a period of one-year, with the diagnosis of breast hypertrophy and who obtained government approval for reduction mammoplasty were invited to participate in this prospective study. After signing an informed consent form, patients were asked to complete several questionnaires at each assessment time: (one week (time one) and one day before surgery (time two) and at one month (time three), six months (time four), and 12 months after surgery (time five)). The questionnaires were the HUI [25-28], and the Breast Reduction Assessment Value and Outcomes (BRAVO) instruments which consist of a set of separate instruments including the SF-36 [29], the MBSRQ-AS [30], and the BRSQ [7,24]. The one-week recall period was used for the HUI, the MBSRQ-AS, and the BRSQ and a four-week recall period was used for the SF-36.

The patients were provided with the questionnaires at their clinic visits and they either completed them while at the clinic or they completed them at home and returned them to the clinic by mail. The patients completed the questionnaires at one week before surgery and at one day before surgery to assess the test-retest reliability of each instrument. The questionnaires were completed at three post-operative time-points to measure change and to assess the stability of change over one-year of follow-up. The Research Ethics Board of McMaster University and St. Joseph's Hospital approved this study.

### Clinical and demographic measures

In addition to completing the quality of life instruments (described in detail below), each patient underwent a physical examination and the baseline information was recorded. Demographic information including age, height, and weight was obtained which permitted the calculation of BMI (kg/m^2^). Other baseline information collected included self-reported bra cup size, diabetes, history of depression, smoking history, shoulder grooving, shoulder pain, back pain, neck pain, breast pain, intertrigo, and history of headaches.

### Generic utility instruments: HUI

The HUI is a well-known health status and quality of life assessment instrument developed as an indirect method of measuring utilities (preferences) in clinical trials and other studies [25-28]. The HUI is a comprehensive, reliable, responsive, and valid multi-attribute utility instrument [25-28]. Responses to the questionnaire are converted using standard algorithms to levels of the Health Utilities Index Mark 2 (HUI2) and Mark 3 (HUI3) multi-attribute health status classification systems. The attribute levels are combined with published scoring functions to calculate utility scores of overall HRQL.

The HUI2 and HUI3 health status classification systems are complementary. Together they provide descriptive measures of ability or disability for health-state attributes, and descriptions of comprehensive health status [28]. The HUI2 is composed of seven attributes or dimensions which are sensation, mobility, emotion, cognition, self-care, pain, and fertility [25-28]. The HUI3 is composed of eight attributes or dimensions: vision, hearing, speech, ambulation, dexterity, emotion, cognition, and pain with five to six levels per attribute [25-28]. A seven-element vector describes the HUI2 comprehensive health state of a patient. Standard HUI questionnaires do not assess HUI2 fertility and, for the purposes of calculating overall HRQL, patients in this study were assumed to have no problems with their fertility. An eight-element vector, one level for each attribute (domain or dimension) of health, describes the HUI3 comprehensive health state for a patient or group of patients. The levels range from highly impaired to normal. For overall health status, the HUI2 and HUI3 utility scales of HRQL are defined such that dead = 0.00 and perfect health = 1.00. The HUI2 describes 24,000 unique health states and the HUI3 describes 972,000 unique health states that are obtained from factorials of the number of levels in each attribute.

Utilities derived from responses to HUI questionnaires may be used to calculate quality adjusted life years (QALYs). QALYs are the measure of effectiveness in cost-utility analysis, a special type of cost-effectiveness analysis for comparing alternative surgical interventions [25-28,31].

### Generic health profile: SF-36

The SF-36 is a multi-purpose, short-form health survey with 36 questions [29]. It is a generic measure, as opposed to one that targets a specific age, disease, or treatment group. Accordingly, the SF-36 has proven useful in surveys of general and specific populations, comparing the relative burden of diseases, and in differentiating the health benefits produced by a wide range of different treatments [29]. The experience to date with the SF-36 has been documented in nearly 4,000 publications; citations for those published in 1988 through 2000 are documented in a bibliography covering the SF-36 and other instruments in the "SF" family of tools [29].

The SF-36 contains multi-function item scales to measure eight domains: physical function (10 items); role physical (4 items); bodily pain (2 items); general health (5 items); vitality (4 items); social functioning (2 items); role emotional (4 items); and mental health (5 items) [29]. The two summary measures of the SF-36 are the physical component summary and the mental component summary [29]. The scores for the multi-function item scales and the summary measures of the SF-36 vary from zero to 100, with 100 being the best possible score and zero being the lowest possible score [29].

### Disease (condition) specific quality of life instruments: MBSRQ-AS and BRSQ

The MBSRQ is a well-validated self-report inventory for the assessment of body image [30]. Body image is conceived as one's attitudinal dispositions toward the physical self. As attitudes, these dispositions include evaluative, cognitive, and behavioral components. The physical self encompasses not only one's physical appearance but also the body's competence or fitness and its biological integrity or health/illness. The MBSRQ is a 69-item self-report inventory for the assessment of self-attitudinal aspects of the body-image construct [30]. The MBSRQ is intended for use with adults and adolescents over the age of 15 years [30]. Two forms of the MBSRQ are available, the full version and the MBSRQ-Appearance Scales (MBSRQ-AS). The full, 69-item version consists of seven factor subscales: 1) appearance evaluation, 2) appearance orientation, 3) fitness evaluation, 4) fitness orientation, 5) health evaluation, 6) health orientation, and 7) illness orientation [30]. There are also three multi-item subscales: 1) the body areas satisfaction scale (BASS), 2) the overweight pre-occupation scales, and 3) the self-classified weight scale [30].

In this study, the shorter version of the MBSRQ-AS was used and only the appearance evaluation subscale was used, because we were concerned with measuring body image. Scores vary from one to five. A high score indicates emphasis on one's looks, attention to one's appearance, and engaging in extensive grooming behaviours. A low score indicates apathy about one's appearance, one's looks are not especially important, and not expending much effort to "look good". High scorers feel mostly positive and satisfied with their appearance; low scorers have a general unhappiness with their physical appearance [30].

The BRSQ lists 13 breast related symptoms and the respondent indicates how much of the time she has the symptoms [7,24]. From this questionnaire, two scores are derived. The first score is the breast symptom summary score (BSS score), which is calculated by taking the mean scores of all 13 items. The BSS score varies from zero to 100, with a high score corresponding to fewer and less severe breast symptoms. For the second score, seven items of the 13-item scale are used to provide the physical symptom count. However, we did not tabulate the physical symptom count for this prospective study, as we were only interested in the overall BRSQ summary score (BSS score). The BRSQ has been validated and has demonstrated good test-retest reliability [7,8,24].

### Scoring of the questionnaires

Scores for the HUI2, the HUI3, and the SF-36 were generated according to algorithms from the developers [32] and the SF-36^® ^Health Survey Manual & Interpretation Guide, [33] respectively. The MBSRQ-AS and the BRSQ were scored according to the algorithm provided by Cash et al and Kerrigan et al, respectively [7,24,30].

### Reliability and validity testing of the HRQL questionnaires

A measure is reliable if it is sound and dependable. Reliability is assessed by tests of repeatability or reproducibility. Reliability is often assessed in terms of agreement between intra-subject test-retest measurements and inter-assessor measurements [34]. There are various ways of assessing reliability of a measure [35]. These can be classified as inter-observer reliability (degree of agreement between different observers) and intra-observer or test-retest reliability (agreement between observations made by the same observer). An intraclass correlation coefficient (ICC) is used in this paper as a statistical measure of agreement for assessing test-retest reliability.

To estimate test-retest reliability, the same HRQL instrument is completed by the same patient on two different occasions. The assumption is that there would be no change in the scorers if there is no substantial change in health status of the patient being measured between the two occasions. The test-retest reliability of patients' responses is extremely important as we were most interested in determining that the difference in scores, between pre- and post-operative times reflected a real change in the patient's health is a result of the surgical intervention. If patient reporting is not reliable then one cannot truly capture the change in health status in patients using HRQL questionnaires.

The reliability of a test is indicated by the reliability coefficient. Reliability is expressed as a number ranging between zero and one; as it approaches zero there is lower reliability and a reliability coefficient close to one indicates higher reliability. In other words, the larger a reliability coefficient is, the more repeatable or reliable the test scores. General guidelines exist for interpreting reliability coefficients. A reliability coefficient value of 0.90 and greater is said to be excellent; a reliability coefficient value of 0.80 to 0.89 is good; a reliability coefficient value of 0.70 to 0.79 is adequate; and a reliability coefficient value below 0.70 may have limited applicability [36].

The validity and reliability of the HUI2, HUI3, and the SF-36 instruments have been demonstrated in various populations [25-29]. The MBSRQ has been validated and some reliability testing has been completed [30]. The BRSQ has been tested for face validity and has undergone test-retest reliability [7,24].

In this study we assessed the test-retest reliability of the HUI2, the HUI3, the MBSRQ-AS, and the BRSQ in patients diagnosed with breast hypertrophy prior to undergoing breast reduction mammoplasty. We did not assess the test-retest reliability of the SF-36 because we had used the four-week recall period for the SF-36. This study also provides some evidence about the concurrent validity of the BRSQ.

### Responsiveness of the HRQL questionnaires

We used two generic and two disease (condition) specific instruments in this prospective study. Generic health status measures seek a broad perspective that is not specifically related to the restricted score of the HRQL of a specific disease or condition. Using a generic instrument has the advantage of allowing comparisons of health status to be made across different diseases and health states [37]. Disease (condition) specific measures focus on the disease or condition being studied, allowing greater sensitivity to intervention-related change compared to generic measures [37]. When deciding to use a generic instrument or a disease (condition) specific instrument to measure HRQL, it is important to consider the responsiveness of a HRQL instrument [37]. There are two major aspects of responsiveness, internal responsiveness and external responsiveness [38]. Internal responsiveness characterizes the ability of a measure to change over a pre-specified timeframe, whereas external responsiveness reflects the extent to which change in a measure relates to a corresponding change in a reference measure of clinical or health status [38]. This study focuses on internal responsiveness.

The effect size index is a statistical measure that can be used as an indicator of internal responsiveness. The mathematical formula for the effect size is the difference (Δ) of mean follow-up assessment scores minus mean baseline assessment score divided by the standard deviation of the baseline scores [39]. Our baseline was one-day before surgery and follow-up was six months after surgery. According to the well-known thresholds set by Cohen, an effect size of less than 0.20 can be considered trivial, an effect size between 0.20 and 0.50 can be considered small, an effect size between 0.50 and 0.80 can be considered moderate, and an effect size greater than 0.80 is considered large [40]. The standardized response mean (SRM) is the mean change scores divided by the standard deviation of the change scores [40].

### Minimum Important Differences (MID)

The minimum important difference is a measure of clinically important or relevant change in health [37]. In other words, the minimum clinically important difference is the minimum level of change of an outcome measure that is considered to be clinically relevant. Drummond reported that differences of 0.03 or greater in mean utility scores were definitely clinically important [41]. This is supported by Grootendorst et al and Horsman et al, who reported that a difference in mean overall HUI scores of 0.03 or more should be considered as clinically important, and by Samsa et al who indicate minimal clinically important differences of HUI overall scores are between 0.02 to 0.04 [28,42,43]. Differences in mean HUI single-attribute utility scores of 0.05 or greater are considered clinically important [28].

There is no rule for determining what constitutes the minimum clinically important difference on the SF-36 subscales [14]. A 10-point change in scores has been suggested as a rule of thumb to apply on 100-point quality of life scales [44]. Minimum important differences have not been reported for the MBSRQ-AS and the BSS score.

### Correlation analyses for assessing redundancy among instruments and concurrent validity of BSS score

Correlation analysis will provide information about the degree of redundancy from measurements using various instruments and evidence about the concurrent validity of the BSS score. Concurrent validity is a form of construct validity [35]. With concurrent validity, a new scale is correlated with another measure thought to be measuring the same construct and both are administered at the same time points [35].

In the current study, the change score of each questionnaire was correlated with the change score of the other questionnaires to assess the degree of redundancy among measures and to assess the concurrent validity of the BSS score. We expected all of the change scores to be positively correlated with each other because they are all scored in a positive direction, measuring improvement.

### Statistical analyses

The patient characteristics were described using frequency distributions and means. The ICC of test-retest reliability was computed using data from one week prior to surgery (time one) and one day prior to surgery (time two) for each HRQL instrument named above. To measure responsiveness, effect size, and standardized response means [39] were calculated for each of the HRQL instruments (HUI2, HUI3, SF-36, MBSRQ-AS, and BRSQ) from one-day before surgery (time two) to six-months after surgery (time four). The Pearson correlation coefficient was calculated using the change score from baseline (one-day before surgery, time two) to six-months after surgery (time four) to assess concurrent validity among the HRQL instruments used in this study. The six-month follow-up was used in the above analyses because there was a higher completion rate than the 12-month follow-up. All statistical analyses were performed using the SPSS statistical software (version 13.01).

## Results

### Completion rates

Fifty-two consecutive patients initially consented to participate in the study. The first patient was enrolled in April 2001 and the last patient was enrolled in May 2002. Of the 52 patients who had initially agreed to participate, 49 patients completed the baseline assessment. Patients did not complete the study for various reasons. One patient could not sufficiently understand English to complete the questionnaires, another patient cancelled her surgery after it had been booked, and one patient decided not to participate. Although 49 patients completed the baseline assessment, some patients did not return their HRQL questionnaires at all time-points despite several telephone calls and mailings (Table 1).

**Table 1:** Number of patients who completed each HRQL instrument at each time point

Time Point	HUI2 and HUI3	SF-36	MBSRQ-AS	BSS Score
1 Week Pre-Op (Time 1)	48	48	47	49
1 Day Pre-Op (Time 2)	47	46	48	49
1 Month Post-Op (Time 3)	42	42	43	44
6 Months Post-Op Time 4	43	40	41	43
1 Year Post-Op Time 5	32	30	30	33

HUI2 = Health Utilities Index Mark 2; HUI3 = Health Utilities Index Mark 3; SF-36 = Short-Form 36; MBSRQ-AS = Multidimensional Body Self Relations Questionnaire Appearance Assessment; BSS Score = Breast Symptom Summary Score

### Clinical and demographic information

The mean age of the patients was 38 years (minimum 20 years; maximum 68 years). The mean BMI was 30.9 kg/m^2 ^(minimum 21.8 kg/m^2^; maximum 49.5 kg/m^2^). Self-reported bra cup sizes ranged from D to H, with 65 percent of the patients having a cup size of DD. Eighteen percent of patients had a history of depression, eight percent experienced frequent headaches, and 12 percent were smokers. Prior to surgery, all of the patients experienced neck pain, 94 percent experienced back pain, 53 percent experienced shoulder grooving, 45 percent experienced shoulder pain, 14 percent had breast pain, and 39 percent had intertrigo. The mean tissue resection weight for the left breast was 757.8 grams and the mean tissue resection weight for the right breast was 822.6 grams.

### Test-Rest reliability

The computed ICC for the HUI2 was 0.86, the HUI3 was 0.84, the MBSRQ-AS was 0.85, and BSS score was 0.87. The HUI2, the HUI3, the BMSRQ-AS, and the BSS score all demonstrated good test-retest reliability.

### Responsiveness

The responsiveness of each instrument is shown in Table 2. The SF-36 physical summary score, the MBSRQ-AS, and the BSS score had a large effect size, therefore, demonstrating high responsiveness. The SF-36 mental component summary and the HUI3 had a moderate effect size and the HUI2 had a small effect size. The SF-36 mental component summary, the HUI2, and the HUI3 had somewhat of a lower responsiveness than the other HRQL instruments used in this study. The standard response means for the measures are of the same magnitude as the effect size.

**Table 2:** Responsiveness of the HRQL instruments used in this study

Measure	Difference	SD at Baseline (1-day pre-op)	SD	ES_1 _	SRM
**HUI2 (n = 41)**	0.06	0.14	0.14	0.45	0.46
**HUI3 (n = 41)**	0.12	0.19	0.17	0.63	0.67
**SF-36 (Physical) (n = 37)**	10.16	8.43	7.45	1.21	1.36
**SF-36 (Mental) (n = 37)**	7.46	11.75	12.63	0.63	0.59
**MBSRQ-AS (n = 40)**	0.86	0.65	0.70	1.32	1.23
**BSS Score (n = 41)**	45.05	13.15	13.74	3.43	3.28

HUI2 = Health Utilities Index Mark 2; HUI3 = Health Utilities Index Mark 3; SF-36 = Short-Form 36; MBSRQ-AS = Multidimensional Body Self Relations Questionnaire Appearance Assessment; BSS Score = Breast Symptom Summary Score; SD= Standard Deviation; ES_1 _= Effect Size (based on Cohen, 1988); SRM = Standardized Response Mean; SRM = Δ/SD(Δ)

ES_1 _= Δ / SD at baseline; Difference (Δ) = mean score at 6 month assessment minus mean score at baseline.

* n's reflect the number of patients who have completed the measure at both time-points (baseline and six months) and hence the difference in numbers from Table 1.

### Minimally Important Differences (MID)

In the current study, the difference identified between the baseline (the day before surgery) and at six-months after surgery was 0.06 for the HUI2 which is twice the minimal important difference identified by Horseman et al [28] (Table 2). For the HUI3, the observed difference was four times the minimal important difference identified above (Table 2). We observed a 10 point increase in the SF-36 physical component summary, which is considered to be of clinical importance (Table 2) [14,44]. However, we did not observe a clinically important increase in the SF-36 mental component summary. The difference observed for the score of the MBSRQ-AS and the BSS score from baseline to six months after surgery was 0.86 and 45.05, respectively (Table 2). Since an effect size of two or more is considered statistically significant (based on the standardized response mean), we believe that this change is clinically important and should be further investigated in other populations.

### Assessing redundancy among measures and concurrent validity of the BSS score

The Pearson's correlations between changes in pairs of HRQL scores are presented in Table 3. Five of the 15 correlations are statistically significant. The HUI2 and HUI3 scores are significantly positively correlated with each other as expected, but scores from HUI2 and HUI3 are not significantly correlated with scores from any other measures. The BSS scores are positively correlated with both SF-36 physical component summary and MBSRQ-AS scores. The MBSRQ-AS scores are positively correlated with the SF-36 mental component summary. The SF-36 physical component summary and the SF-36 mental component summary are negatively correlated. Moderate or better associations were observed for HUI2 emotion with SF-36 mental component summary (r = 0.489, p = 0.003) and MBSRQ-AS (r = 0.618, p < 0.001), for the HUI3 emotion with SF-36 mental component summary (r = 0.501, p = 0.002), and for HUI3 pain with SF-36 physical component summary (r = 0.412, p = 0.013).

**Table 3:** Correlations between changes in the HRQL scores

	HUI3	SF-36 (Physical)	SF-36 (Mental)	MBSRQ-AS	BSS Score
**HUI2**					
Pearson Correlation	0.625**	0.135	0.295	0.317	0.221
p-value (2-tailed)	<0.001	0.431	0.081	0.053	0.170
n	41	36	36	38	40
**HUI3**					
Pearson Correlation		0.128	0.273	0.127	0.198
p-value (2-tailed)		0.458	0.107	0.446	0.222
n		36	36	38	40
**SF-36 (Physical)**					
Pearson Correlation			-0.515**	-0.187	0.359*
p-value (2-tailed)			0.001	0.289	0.029
n			37	34	37
**SF-36 (Mental)**					
Pearson Correlation				0.484**	0.147
p-value (2-tailed)				0.004	0.386
n				34	37
**MBSRQ-AS**					
Pearson Correlation					0.481**
p-value (2-tailed)					0.002
N					40

n's reflect the number of patients who have completed the measure at both time-points (baseline and six months) and hence the difference in numbers from Table 1.

** Correlation is significant at the 0.01 level (2 tailed)

* Correlation is significant at the 0.05 level (2 tailed)

HUI2 = Health Utilities Index Mark 2; HUI3 = Health Utilities Index Mark 3; SF-36 = Short-Form 36; MBSRQ-AS = Multidimensional Body Self Relations Questionnaire Appearance Assessment; BSS Score = Breast Symptom Summary Score

## Discussion

This study included patients with the diagnosis of breast hypertrophy who had obtained government approval for reduction mammoplasty. In our geographical area (Ontario, Canada), in contrast to other jurisdictions, for example, Nova Scotia, Canada [18] and the United States [19], the approval for provincial coverage for reduction mammoplasty is almost always granted if the patient has a bra cup size of D or larger and is experiencing physical symptoms.

A number of previous studies have reported that women who suffer from breast hypertrophy frequently present with heightened body image dissatisfaction [45-48]. In Canada, when plastic surgeons are faced with lawsuits, it is most commonly from breast surgery and when they are sued by patients following a breast reduction surgery it is usually due to the appearance of the breast or scarring [49]. Body image is conceived as one's attitudinal dispositions toward the physical self. As attitudes, these dispositions include evaluative, cognitive, and behavioral components. A study of the preoperative body image concerns of breast reduction patients found increased dissatisfaction with both their overall body image and breast size [46]. In response to their excessive breast size, patients reported extreme embarrassment in public areas and social settings and significant avoidance of physical activity [46]. Several previous studies on patients with breast hypertrophy have used the MBSRQ-AS to measure body image and have found that women with breast hypertrophy had low scores on the MBSRQ-AS suggesting dissatisfaction with their overall body image [7,8,24,46].

Patients completed the HUI2, the HUI3, and the BRAVO instruments (the SF-36, the MBSRQ-AS, and the BRSQ) at one week and one day before surgery to measure the test-retest reliability of each instrument and at one, six, and 12 months after surgery to measure change in HRQL following breast reduction mammoplasty. The methodology used in this prospective study may interest those who wish to sponsor, design, or implement future HRQL studies in breast reduction surgery or other areas of plastic surgery.

Of the 52 patients who had initially agreed to participate, 49 patients completed the baseline assessment. Despite multiple reminders, 30 patients completed all of the HRQL questionnaires at the 12-month follow up. This equates to a compliance rate of 57.7 percent. The response rate in this study is comparable to response rates obtained in previous studies on HRQL in patients with breast hypertrophy. For instance, several authors have reported response rates ranging from 32.5 percent to 80 percent [5,11,12,14,16]. For future studies, it may be helpful to understand why patients may not complete all of the requirements of a research study. The burden of completing multiple questionnaires may have limited our rate of compliance at one year. Patients who withdrew consent from one multi-centre trial reported interference with work, lack of time, complicated and cumbersome record keeping requirements, difficult study medicine regimens, and difficulty scheduling appointments due to a lack of flexibility on the part of the study personnel [50]. In the above study, the matched patients who completed all of their follow up reported that remuneration, commitment to finish, and the belief that the study was important motivated them to fully complete the study [50]. Based on existing guidelines for self-administered questionnaires, the questionnaires used in the present study exceeded the 12-page upper limit recommendation [51].

To measure the test-retest reliability of each instrument, scores were obtained for each instrument using the recommended algorithms and the ICC was computed from these scores. We found that all HRQL instruments demonstrated good reliability, which reinforces previous reliability testing of the HUI2, HUI3, SF-36, MBSRQ-AS, and BSS score.

It is extremely important that there is low within-patient variability in stable patients, relative to the magnitude of change that is predicted following the intervention, while answering the various questions on quality of life questionnaires in surgical outcome studies. Absence of reliable reporting will reduce the ability of measures to assess the effectiveness of surgery. For the present study, the one-week interval (time one and time two) was chosen to assess patient reporting as it was not long enough for other adverse events to intervene and change the health status but appropriate to avoid recall bias.

Marx et al noted that if multiple questionnaires were administered, each consisting of numerous items, the effect of memory may be minimized and the effect of memory may be greater if only a single questionnaire was used [52]. In the present study, four HRQL questionnaires were administered, each with multiple questions so the effect of a patient's memory is likely to be limited, therefore not biasing the responses.

The SF-36 physical component summary, the MBSRQ-AS, and the BSS score showed high responsiveness. The SF-36 mental component summary, the HUI2, and the HUI3 had a lower responsiveness summary statistics than the other HRQL instruments used in this study but all three instruments were able to detect clinically important changes in overall HRQL scores. The HUI3 showed a moderate effect size and detected a clinically important reduction in pain scores. All of the statistically significant correlations are positive except for the SF-36 physical component summary with the SF-36 mental component summary. The negative correlation may be a function of the problem with the algorithms for calculating SF-36 physical and mental component summary scores described in the published literature including reports by Simon et al [53] and Cunningham et al [54]. This study confirms evidence of concurrent validity for the BSS score as the change in BSS score is highly correlated with the SF-36 and other HRQL measures [19]. The HUI scores appear to provide unique information, as they were not correlated with the other measures. There were moderate or stronger correlations of HUI single-attribute utility scores, for emotion and pain, with the SF-36 and MBSRQ-AS.

This study demonstrates that patient reporting using the HUI2, the HUI3, the MBSRQ-AS, and the BSS score are reliable in a sample of patients diagnosed with breast hypertrophy who had breast reduction mammoplasty. All instruments were equally reliable. The HUI is the only preference-based instrument and it was shown to be responsive. The two disease (condition) specific instruments were the most responsive of all the HRQL instruments used

Having established the reliability and responsiveness of two generic (HUI and SF-36) instruments and two disease (condition) specific (MBSRQ-AS and BSS score) instruments, and the concurrent validity of BSS score, the focus moves onto the clinical and policy implications of the prospective study by addressing the following question: Can the improvement in HRQL derived from breast reduction surgery be measured quantitatively? Research is underway to address four specific issues: 1) identifying health attributes affected most frequently in breast hypertrophy patients and describing the extent of the observed morbidity; 2) assessing the health status and HRQL of patients in short, intermediate, and long time periods after reduction mammoplasty (i.e. one, six, and 12 months); 3) determining if there is a relationship between tissue resection weight and changes in health status and HRQL; and 4) determining if there is a relationship BMI and changes in health status and HRQL to address the ongoing BMI discrimination by third party payers.

## List of abbreviations

BRAVO Breast Reduction Assessment Value and Outcomes

BRSQ Breast Related Symptoms Questionnaire

BSS Score Breast Symptom Summary Score

HRQL Health-Related Quality of Life

HUI Health Utilities Index

HUI2 Health Utilities Index Mark 2

HUI3 Health Utilities Index Mark 3

ICC Intraclass Correlation Coefficient

MBSRQ-AS Multidimensional Body Self Relations Questionnaire Appearance Assessment

MID Minimally Important Differences

SF-36 Short Form-36

SRM Standardized Response Mean

## Authors' contributions

AT: Conception of the study, design of the study, acquisition of data, drafting of the manuscript. SS: Drafting of the manuscript. KV: Study coordination, acquisition of data, critical review of the manuscript. ED: Design of the study, statistical analysis, critical review of the manuscript. BF: Study design, critical review of the manuscript. All authors have read and approved the final manuscript.
